# Supplementary material for: Small signal analysis for the characterization of organic electrochemical transistors
Source: Nat Commun. 2024 Sep 1;15:7606. doi: 10.1038/s41467-024-51883-9 (PMC11366767; doi:10.1038/s41467-024-51883-9)
Supplement: Supplementary file 1 — Supplementary Information [file 41467_2024_51883_MOESM1_ESM.docx]

**Small Signal Analysis for the Characterization of Organic Electrochemical Transistors**

Youngseok Kim,^1*^ Joost Kimpel,^1^ Alexander Giovannitti,^1^ Christian Müller^1*^

^1^ Department of Chemistry and Chemical Engineering, Chalmers University of Technology, 412 96 Göteborg, Sweden

* ykim@chalmers.se; christian.muller@chalmers.se


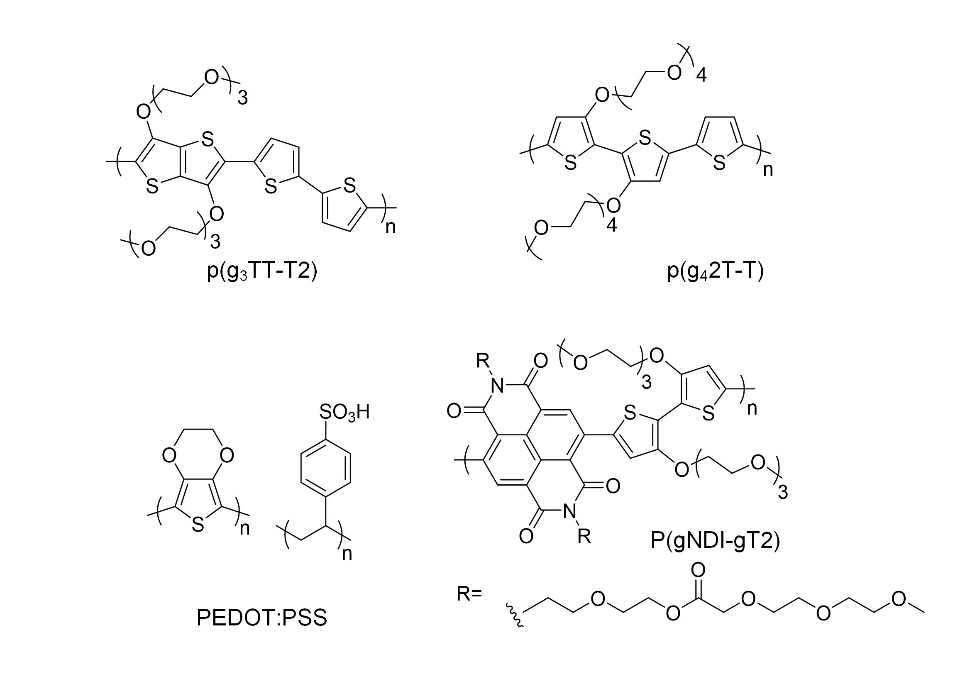


**Fig. S1. Chemical structures used in this study.** Repeat units of the polymers p(g_3_TT-T2),
p(g_4_2T-T), PEDOT:PSS and p(gNDI-gT2).


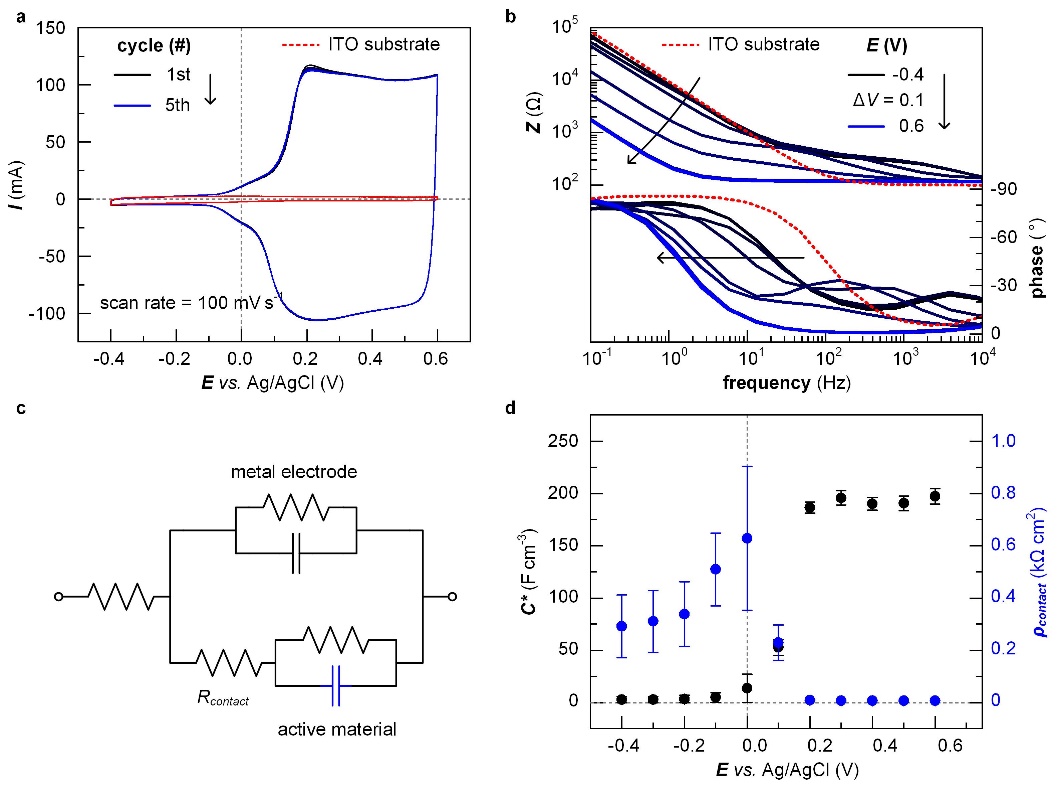


**Fig. S2. Electrochemical characterization of p(g_3_TT-T2).** (a) Cyclic voltammetry of thin films for 5 cycles. (b) Impedance *Z* (upper panel) and phase curves (lower panel) as a function of the offset potential *E* *vs.* Ag/AgCl from -0.4 to +0.6 V. (c) Equivalent circuit for the circuit fitting, and (d) extracted volumetric capacitance $C^{*}$and contact resistivity $\rho_{contact}=R_{contact}/contact area$ as a function of *E*.


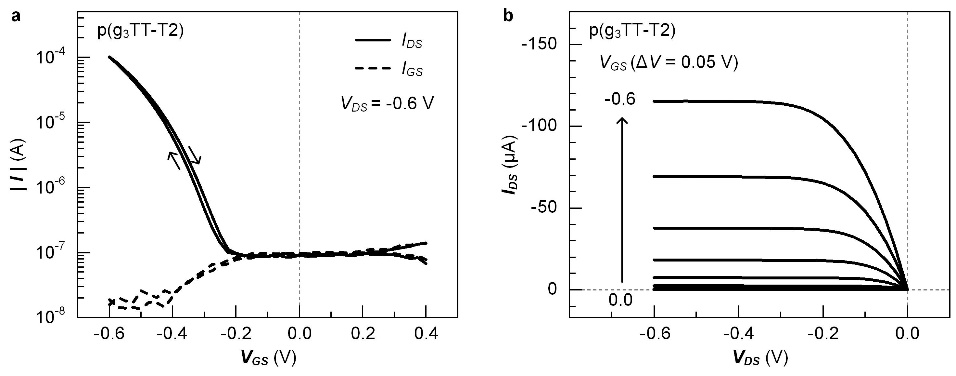


**Fig. S3. Transfer and output curves** of p(g_3_TT-T2) based OECTs obtained by the conventional characterization method.


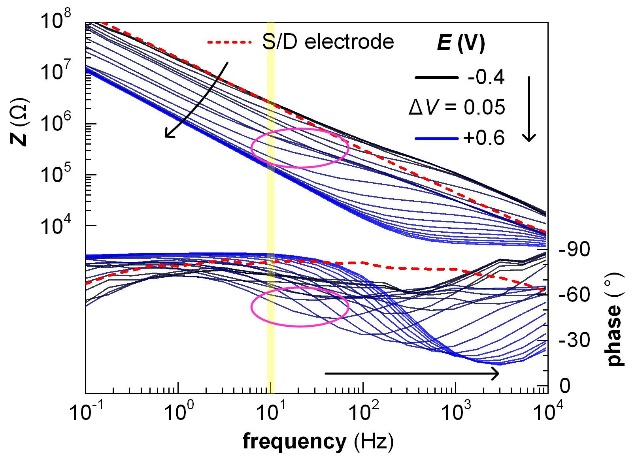


**Fig. S4. EIS impedance and phase curves which were characterized with OECT devices.** The measurement condition is identical with those shown in Fig. S2b.


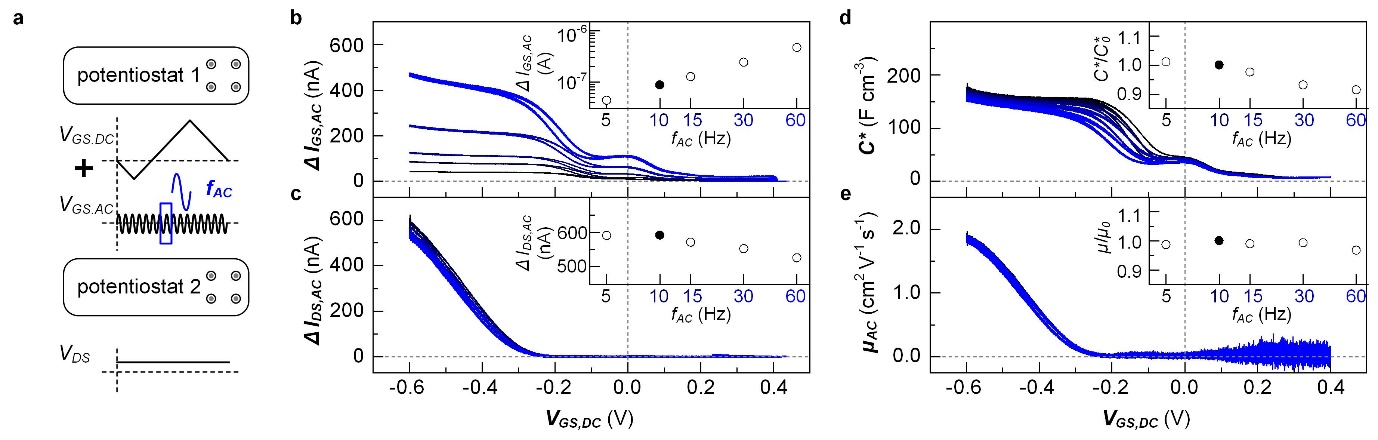


**Fig. S5. Impact of the frequency** $\boldsymbol{f}_{\boldsymbol{AC}}$ **of the gate input potential.** (a) Schematic of measurement. $f_{AC}$ value was scanned from 5 (black) to 60 Hz (blue). Traces of (b) $\Delta I_{GS,AC}$ and (c) $\Delta I_{DS,AC}$. Insets depict the amplitude at $V_{GS,DC}$ = −0.6 V. Due to the decreased impedance at higher frequencies, $\Delta I_{GS,AC}$ value is linearly increased. Traces of (d) $C^{*}$ and (e) $\mu_{AC}$ as a function of $V_{GS,DC}$ (Insets: relative $C^{*}$ and $\mu_{AC}$ with respect to the value at $f_{AC}$= 10 Hz).


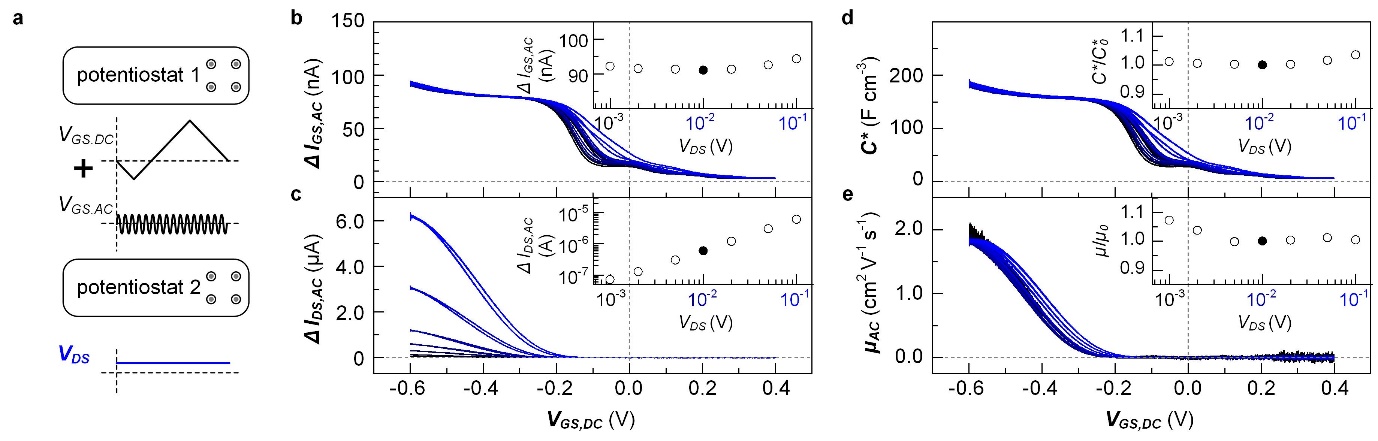


**Fig. S6. Impact of the amplitude of** $\boldsymbol{V}_{\boldsymbol{DS}}$**.** (a) Schematic of measurement. Amplitude of $V_{DS}$ was scanned from 1 (black) to 100 mV (blue). Traces of (b) $\Delta I_{GS,AC}$ and (c) $\Delta I_{DS,AC}$. Insets depict the amplitude at $V_{GS,DC}$ = −0.6 V. Since the device is in the linear regime, $\Delta I_{DS,AC}$ shows a linear correlation with the amplitude of $V_{DS}$. Traces of (d) $C^{*}$ and (e) $\mu_{AC}$ as a function of $V_{GS,DC}$ (Inset: relative $C^{*}$ and $\mu_{AC}$ with respect to the value at $V_{DS}$ = 10 mV).


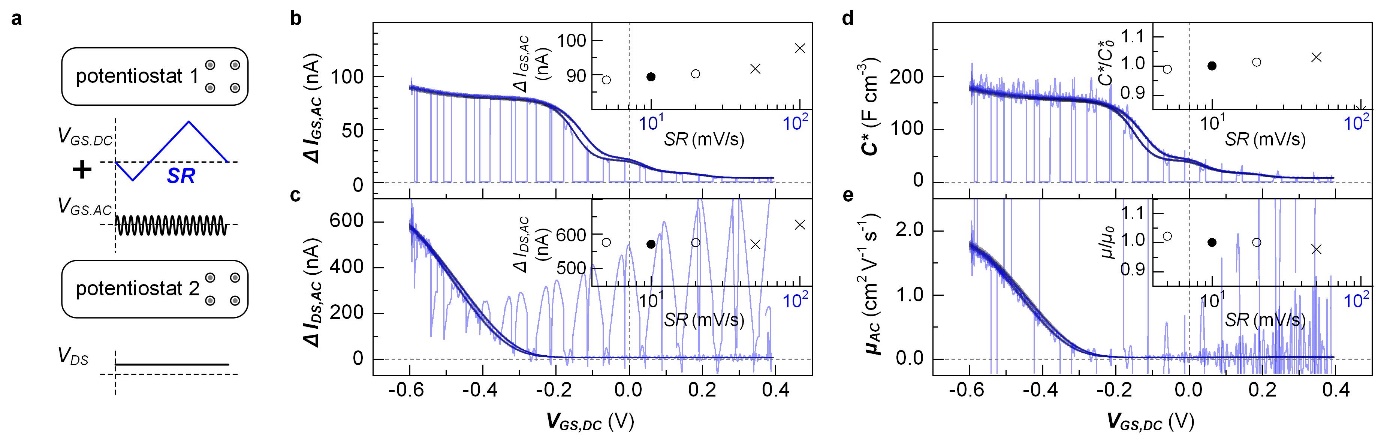


**Fig. S7. Impact of the scan rate** $\boldsymbol{SR}$ **of** $\boldsymbol{V}_{\boldsymbol{GS,DC}}$**.** (a) Schematic of measurement. $SR$ value was scanned from 5 (black) to 100 mV s^-1^ (blue). Traces of (b) $\Delta I_{GS,AC}$ and (c) $\Delta I_{DS,AC}$. Inset depicts the amplitude at $V_{GS,DC}$ = −0.6 V as a function of $SR$. At $SR$ > 50 mV s^-1^, the extracted results are not clear due to the signal interference at $f_{AC}$ = 10 Hz in frequency domain from the fast linear sweep potential. Traces of (d) $C^{*}$ and (e) $\mu_{AC}$ as a function of $V_{GS,DC}$ (Insets: relative $C^{*}$ and $\mu_{AC}$ with respect to the value at $SR$ = 10 mV s^-1^).

**SI Note 1. Small signal analysis of various OMIEC materials**

All materials were characterized with a gate potential window of 1 V (−0.6 🡪 +0.4 V for p(g_4_2T-T); −0.2 🡪 +0.8 V for PEDOT:PSS; −0.8 🡪 +0.2 V and −0.5 🡪 +0.5 V for p-/n-type operation of p(gNDI-gT2)) and a $V_{DS}$ = 0.01 V for p(g_4_2T-T) and PEDOT:PSS, and $V_{DS}$ = +0.1/−0.1 V for p-/n-type operation of p(gNDI-gT2).

In case of OECTs based on p(g_4_2T-T), $I_{GS}$ and $I_{DS}$ increased with $V_{GS,DC}$, showing an on-current value near 20 μA and an on-off current ratio of 10^3^ (**Fig. S8a-c**). All device characteristics are comparable to those of p(g_3_TT-T2) based OECTs (**Fig. S8d-i**). $C^{*}$ increased for $V_{GS,DC}$ < 0.15 V, revealing a shift in onset potential by 0.25 V compared to that of devices based on p(g_3_TT-T2), which is due to the lower ionization energy $IE$ of the former ($IE$ = 4.3 and 4.5 eV for p(g_4_2T-T)^1^ and p(g_3_TT-T2)^2^). $\mu_{AC}$ gradually increased as $V_{GS,DC}$ < $V_{onset}$ − 0.35 V, while a potential difference of −0.2 V is required in case of p(g_3_TT-T2) based OECTs (see **Figs. 3c** and **3f**). In case of both materials, we explain the difference between the onset potentials of $C^{*}$ and $\mu_{AC}$ with the structural changes that occur during oxidation, leading to the formation of a conducting network at which point the mobility increases.^3^ Also, the absence of a peak in $\Delta I_{GS,AC}^{'}$ (data not shown), which was observed near the onset potential of $C^{*}$for p(g_3_TT-T2) (see **Fig. 2g**), indicates a lower contact resistance in case of p(g_4_2T-T) based devices. OECTs based on p(g_4_2T-T) yield a maximum $\left[ \mu C^{*} \right]_{AC}$ = 220 F cm^−1^ V^−1^ s^−1^ a $\mu_{AC}$ = 1.25 cm^2^ V^−1^ s^−1^, which is in good agreement with previously reported values.^4,5^


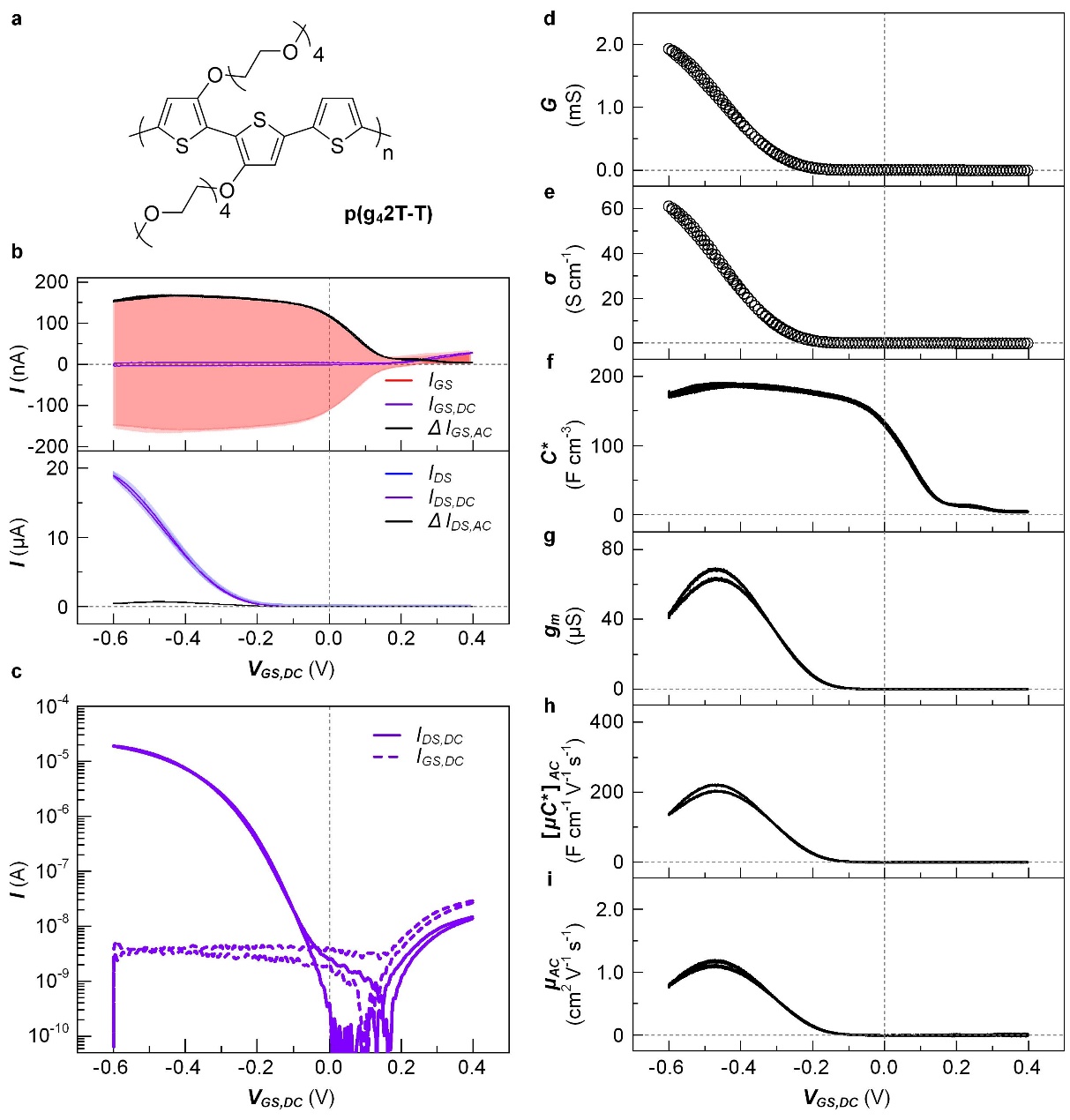


**Fig. S8. Characterization of p(g_4_2T-T)** **through small signal analysis.** (a) Chemical structure of p(g_4_2T-T). (b) $I_{GS}$ (upper panel) and $I_{DS}$ (lower panel) as a function of $V_{GS,DC}$. (c) $I_{DS,DC}$ and $I_{G,DC}$ as a function of $V_{GS,DC}$. Traces of (d) $G$ (e) $\sigma$, (f) $C^{*}$, (g) $g_{m}$, (h) $\left[ \mu C^{*} \right]_{AC}$, and (i) $\mu_{AC}$ as a function of $V_{GS,DC}$.

The depletion mode operation of PEDOT:PSS based OECTs (**Fig. S9a**) is evidenced by the device characteristics (see $I_{GS}$ and $I_{DS}$ vs. $V_{GS,DC}$ in **Fig. S9b**) showing an on-current of 60 μA at $V_{GS,DC}$ = 0 V, and a near zero value at $V_{GS,DC}$ > +0.7 V with an on-off current ratio of 10^4^ (**Fig. S9c**). $\Delta I_{GS,AC}$ and corresponding $C^{*}$ values do not significantly change in the potential window from −0.2 to +0.8 V, but decrease at $V_{GS,DC}$ > 0.8 V (data not shown). Above 0.8 V, however, the device behavior becomes unstable, which we explain with permanent degradation due to excessive volume expansion as a result of cation uptake accompanied by the electrolyte solvent (water).^6^ Maximum values of $\left[ \mu C^{*} \right]_{AC}$ = 351 F cm^−1^ V^−1^ s^−1^ and $\mu_{AC}$ = 4.0 cm^2^ V^−1^ s^−1^ were achieved at $V_{GS,DC}$ = +0.2 V, in agreement with previous reports.^7–10^


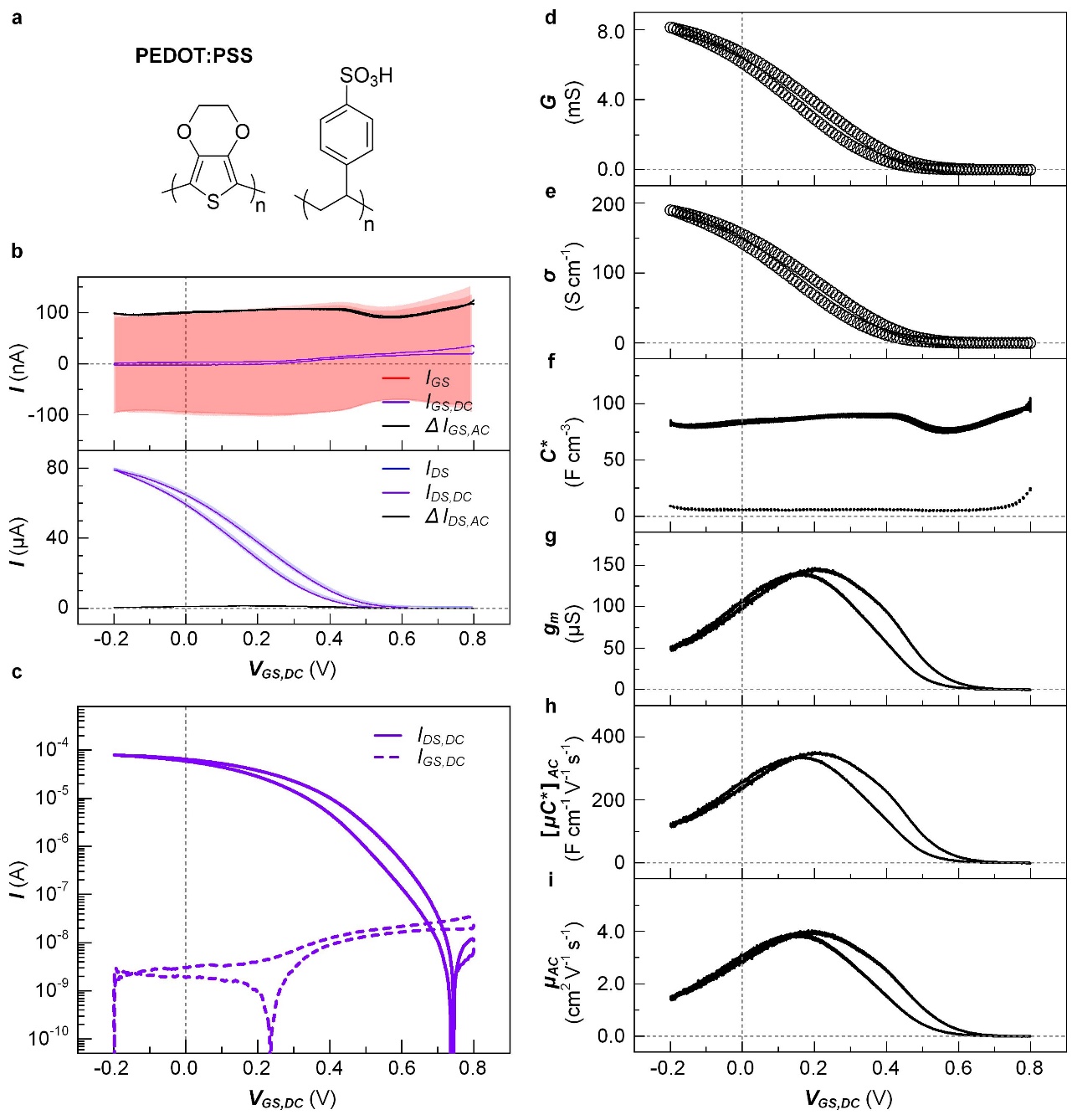


**Fig. S9. Characterization of PEDOT:PSS** **through small signal analysis.** (a) Chemical structure of PEDOT and PSS. (b) $I_{GS}$ (upper panel) and $I_{DS}$ (lower panel) as a function of $V_{GS,DC}$. (c) $I_{DS,DC}$ and $I_{G,DC}$ as a function of $V_{GS,DC}$. Traces of (d) $G$ (e) $\sigma$, (f) $C^{*}$, (g) $g_{m}$, (h) $\left[ \mu C^{*} \right]_{AC}$, and (i) $\mu_{AC}$ as a function of $V_{GS,DC}$.

Prior to small signal analysis of OECTs based on the ambipolar material p(gNDI-gT2), we conducted EIS, cyclic voltammetry (CV) and conventional OECT characterization because of the complexity of mixed p-type and n-type operation. Cyclic voltammograms (**Fig. S10a**) confirm that the material shows a combination of both types of electrochemical response at $E$ > +0.5 V and $E$ < −0.15 V, with two distinct volumetric capacitance values of $C^{*}$ = 150 F cm^−3^ at $E$ = +0.8 V and $C^{*}$ = 225 F cm^−3^ at $E$ = −0.5 V (**Fig. S10b-d**), which are associated with hole-anion and electron-cation accumulation, respectively. The output characteristics of p-type operation devices (**Fig. S11a**) exhibit clear linear and saturation regimes, which are evident for $V_{GS}$ < −0.4 V, while n-type operation is observed for $V_{GS}$ > −0.4 V and $V_{DS}$ < −0.3 V (see the onset potential during reduction in **Fig. S10a**).^11^ Instead, output characteristics for n-type operation also show linear and saturation mode operation at $V_{GS}$ > −0.2 V (**Fig. S11b**), resulting in ambipolar behavior (**Fig. S11c** and **d**).


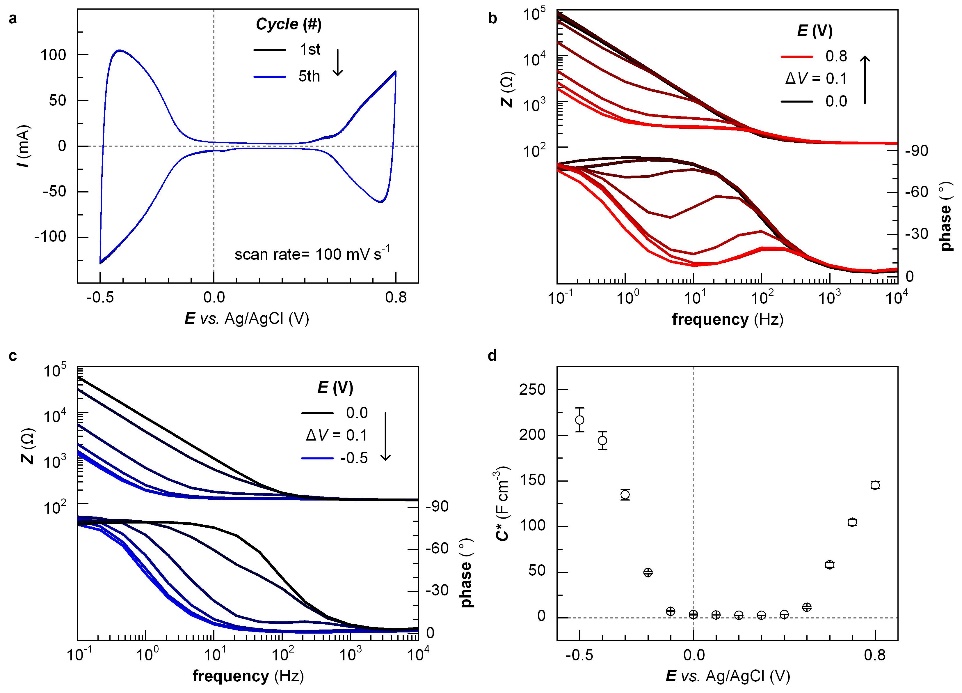


**Fig. S10. Electrochemical characterization of p(gNDI-gT2).** (a) Cyclic voltammetry of thin films for 5 cycles. Impedance $Z$ (upper panel) and phase curves (lower panel) as a function of the offset potential $E$ *vs.* Ag/AgCl (b) from 0.0 (black) to +0.8 V (red), and (c) from 0.0 (black) to −0.5 V (blue). (d) Extracted volumetric capacitance $C^{*}$ as a function of $E$.


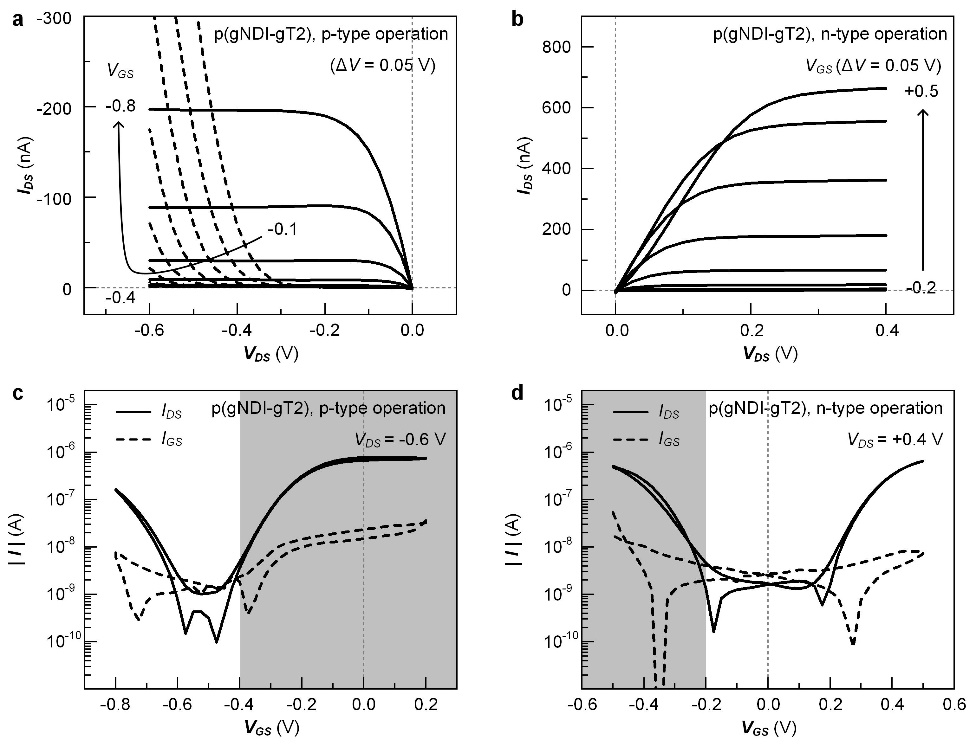


**Fig. S11. (a-b) Output and (c-d) transfer curves** of p(gNDI-gT2)-based OECTs, which were obtained by the conventional characterization method for (a, c) p-type and (b, d) n-type operation. In the transfer curves, the electronic conduction by opposite charge carriers is remarked with a gray color.

In case of p-type operation, both $I_{GS}$ and $I_{DS}$ gradual increase as $V_{GS,DC}$ changes from −0.4 to −0.6 V (see **Fig. S12a-b**). In contrast to the transfer curves recorded in the saturation regime (see **Fig. S11c**), only the electronic current by holes is monitored at $V_{GS,DC}$ < 0, attributed to the low amplitude of $V_{DS}$ of +0.1 V (**Fig. S12c**). Further, $I_{GS,DC}$ and $I_{DS,DC}$ agree with a transfer curve recorded using the conventional method. Values of $G$ = 3.7 μS and $\sigma$ = 135 mS cm^-1^ are lower than those reported for other p-type materials, despite of a high $C^{*}$ = 125 F cm^−3^ at $V_{GS,DC}$ = −0.8 V. Maximum $g_{m}$ and $\left[ \mu C^{*} \right]_{AC}$ values of 2.27 μS and 0.84 F cm^−1^ V^−1^ s^−1^ are achieved at $V_{GS,DC}$ = −0.7 V, while the mobility reaches a value of $\mu_{AC}$= 0.0076 cm^2^ V^−1^ s^−1^ at $V_{GS,DC}$ = −0.7 V.


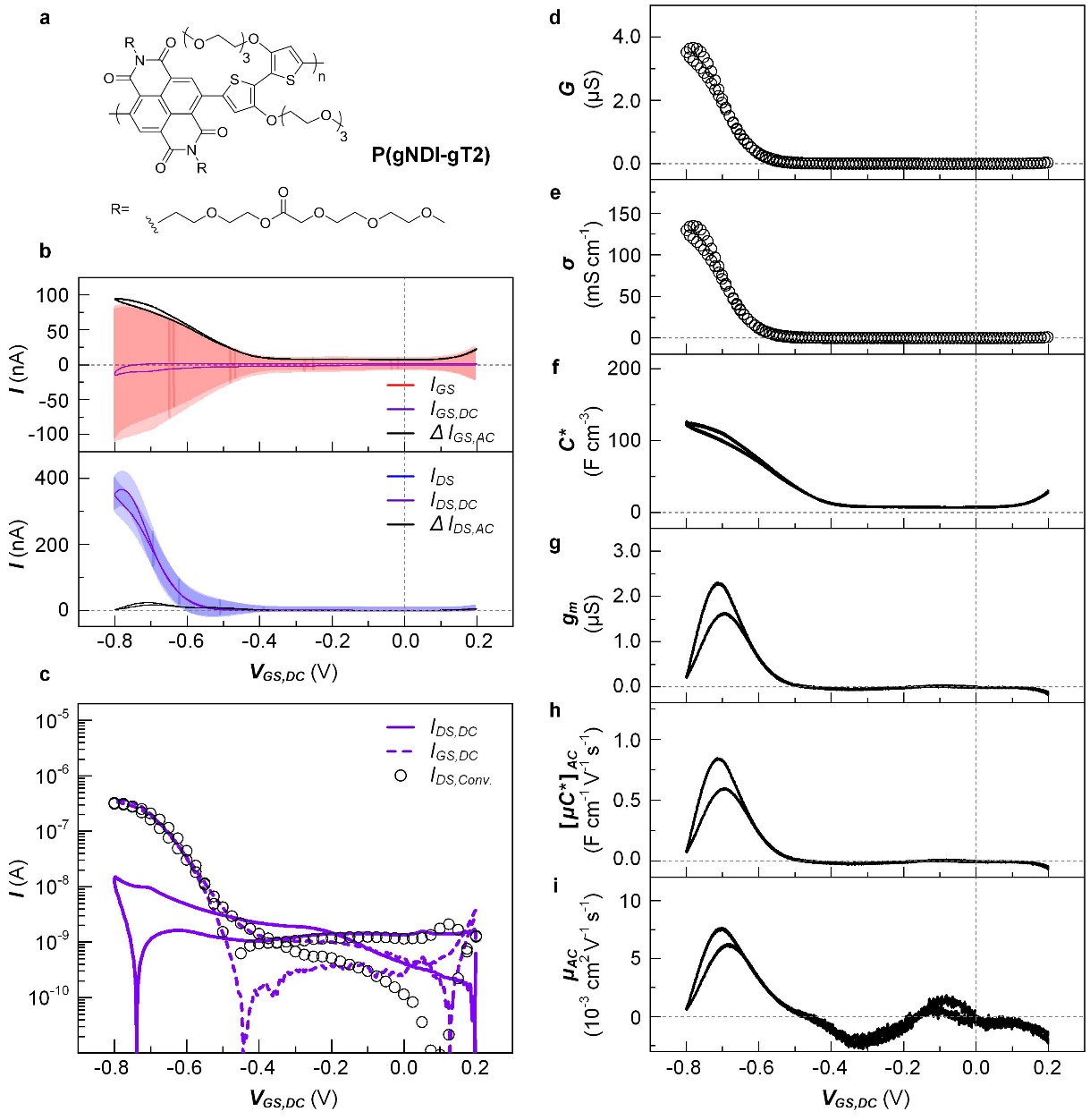


**Fig. S12. Characterization of p(gNDI-gT2) in p-type operation through small signal analysis.** (a) Chemical structure of p(gNDI-gT2). (b) $I_{GS}$ (upper panel) and $I_{DS}$ (lower panel) as a function of $V_{GS,DC}$. (c) $I_{DS,DC}$ and $I_{G,DC}$ as a function of $V_{GS,DC}$. Traces of (d) $G$ (e) $\sigma$, (f) $C^{*}$, (g) $g_{m}$, (h) $\left[ \mu C^{*} \right]$, and (i) $\mu_{AC}$ as a function of $V_{GS,DC}$.

In case of n-type operation, the electrochemical and electrical response occurred for positive $V_{GS,DC}$ (+0.05 and +0.15 V, respectively) (**Fig. S13a-c**). The negative slope of $I_{DS}$ above $V_{GS,DC}$ = +0.36 V resulted in a negative slope in $G$ and $\sigma$ traces (**Fig. S13d-e**). Even though, $C^{*}$ values gradually increased to 200 F cm^−3^ (**Fig. S13f**), negative $g_{m}$, $\left[ \mu C^{*} \right]$, and $\mu$ values were obtained (**Fig. S13g-h**), which are the main characteristics of anti-ambipolar transistors that can be employed for the construction of neuromorphic devices,^12^ frequency doubler circuits^13^ and ternary logic circuits.^14^ Considering the inversion of $I_{DS}$ above $V_{GS,DC}$ = −0.4 V, as shown in the output characteristic curves (see **Fig. S11b**), we attribute the negative values of $g_{m}$, $\left[ \mu C^{*} \right]$, and $\mu$ to increased contact resistance at $V_{GS,DC}$ > 0.36 V.^15^ Note that the apparent negative $\mu$ value does not imply opposite direction of charge-carrier motion (**Fig. S13i**), but may arise due to the parasitic contact resistance, opening of a hard Coulomb gap,^16^ and/or band-to-band tunneling behavior^17^ etc.


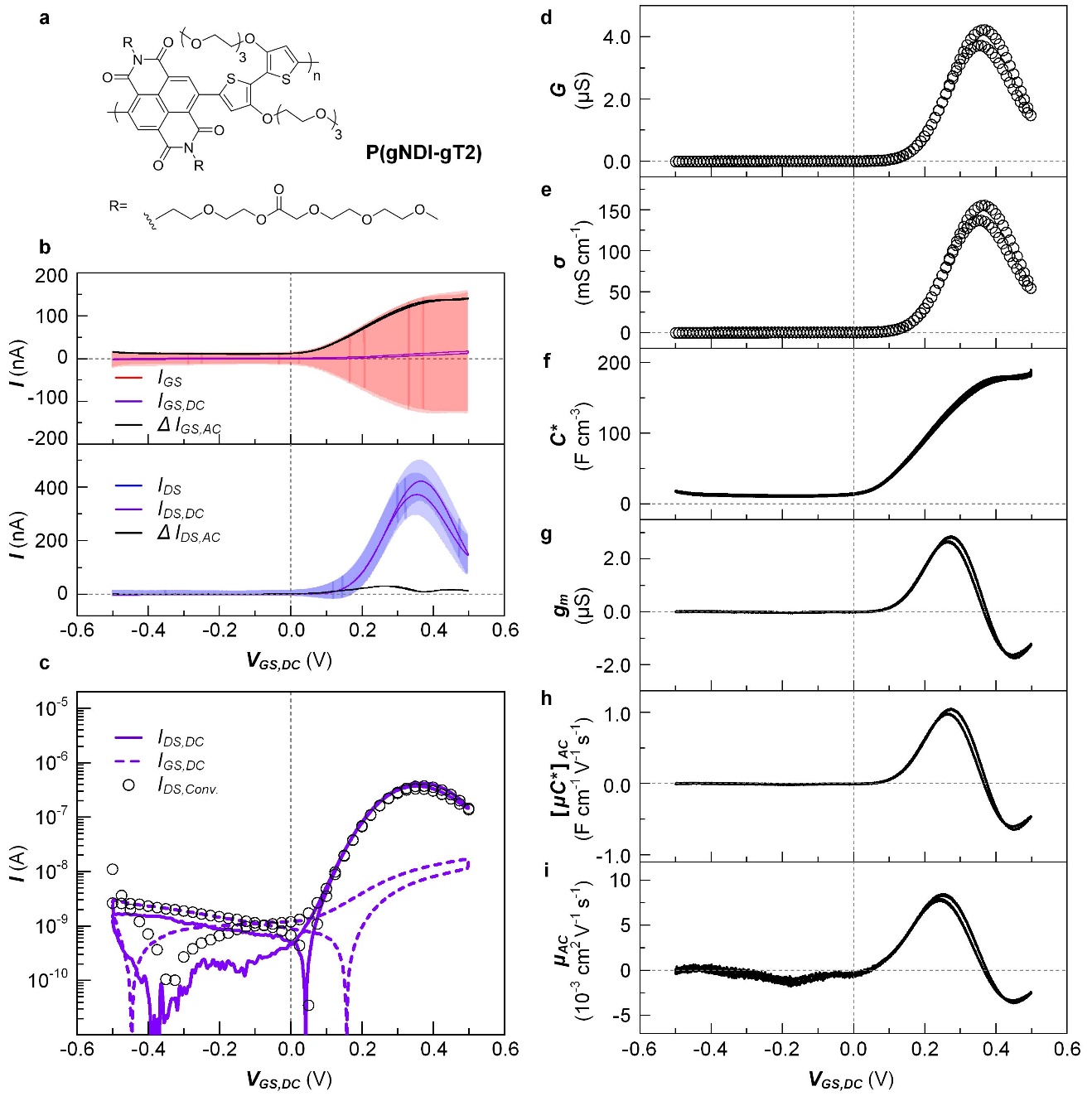


**Fig. S13. Characterization of p(gNDI-gT2) in n-type operation through small signal analysis.** (a) Chemical structure of p(gNDI-gT2). (b) $I_{GS}$ (upper panel) and $I_{DS}$ (lower panel) as a function of $V_{GS,DC}$. (c) $I_{DS,DC}$ and $I_{G,DC}$ as a function of $V_{GS,DC}$. Traces of (d) $G$ (e) $\sigma$, (f) $C^{*}$, (g) $g_{m}$, (h) $\left[ \mu C^{*} \right]_{AC}$, and (i) $\mu_{AC}$ as a function of $V_{GS,DC}$.

**Derivation of Equation 5**

| $C=\frac{dq}{dV_{GS}}$ | ∵ definition of the capacitance $C\equiv dq/dV$, with $V$ = $V_{GS}$ = $A\sin\left( 2\pi ft \right)$; input signal through the gate electrode. |
| --- | --- |
| $=\frac{dq}{dt}\cdot\frac{dt}{dV_{GS}}$ |  |
| $=\frac{I_{GS,AC}^{''}}{2\pi f A\cos\left( 2\pi ft \right)}$ | ∵ definition of the electric current $I\equiv dq$/$dt$, with $I=I_{GS,AC}^{''}$; imaginary component of output measured current through the gate electrode,  and $dV_{GS}$/$dt$ = $2\pi f A\cos\left( 2\pi ft \right)$ |
| $=\frac{\Delta I_{GS,AC}^{''}}{2\pi f \Delta V_{GS,AC}}$ | ∵ expression in frequency domain |
| $\therefore C^{*}=\frac{\Delta I_{GS,AC}^{''}}{2\pi f \Delta V_{GS,AC}\cdot vol}$ | ∵$C^{*}$ = $C/vol$ |

**Supplementary References**

1. Xu, K. *et al.* Ground-state electron transfer in all-polymer donor–acceptor heterojunctions. *Nat. Mater.* **19**, 738–744 (2020).

2. Kimpel, J. *et al.* High-Mobility Organic Mixed Conductors with a Low Synthetic Complexity Index via Direct Arylation Polymerization. *Chem. Sci.* **15**, 7679–7688 (2024).

3. LeCroy, G. *et al.* Role of aggregates and microstructure of mixed-ionic–electronic-conductors on charge transport in electrochemical transistors. *Mater. Horiz.* **10**, 2568–2578 (2024).

4. Mone, M. *et al.* Mechanically Adaptive Mixed Ionic-Electronic Conductors Based on a Polar Polythiophene Reinforced with Cellulose Nanofibrils. *ACS Appl. Mater. Interfaces* **15**, 28300–28309 (2023).

5. Impact of Oligoether Side-Chain Length on the Thermoelectric Properties of a Polar Polythiophene | ACS Applied Electronic Materials. https://pubs.acs.org/doi/full/10.1021/acsaelm.3c00936.

6. Paleti, S. H. K. *et al.* Impact of doping on the mechanical properties of conjugated polymers. *Chem. Soc. Rev.* **53**, 1702–1729 (2024).

7. Kim, Y. *et al.* Strain-Engineering Induced Anisotropic Crystallite Orientation and Maximized Carrier Mobility for High-Performance Microfiber-Based Organic Bioelectronic Devices. *Adv. Mater.* **33**, 2007550 (2021).

8. Inal, S., Malliaras, G. G. & Rivnay, J. Benchmarking organic mixed conductors for transistors. *Nat. Commun.* **8**, 1767 (2017).

9. Rivnay, J. *et al.* Structural control of mixed ionic and electronic transport in conducting polymers. *Nat. Commun.* **7**, 11287 (2016).

10. Keene, S. T. *et al.* Enhancement-Mode PEDOT:PSS Organic Electrochemical Transistors Using Molecular De-Doping. *Adv. Mater.* **32**, 2000270 (2020).

11. Giovannitti, A. *et al.* N-type organic electrochemical transistors with stability in water. *Nat. Commun.* **7**, 13066 (2016).

12. Harikesh, P. C. *et al.* Ion-tunable antiambipolarity in mixed ion–electron conducting polymers enables biorealistic organic electrochemical neurons. *Nat. Mater.* **22**, 242–248 (2023).

13. Luo, Z. *et al.* Negative transconductance in multi-layer organic thin-film transistors. *Nanotechnology* **30**, 02LT01 (2018).

14. Lee, C. *et al.* Systematic Control of Negative Transconductance in Organic Heterojunction Transistor for High-Performance, Low-Power Flexible Ternary Logic Circuits. *Small* **17**, 2103365 (2021).

15. Liu, Y. *et al.* Vertical Charge Transport and Negative Transconductance in Multilayer Molybdenum Disulfides. *Nano Lett.* **17**, 5495–5501 (2017).

16. Xu, K. *et al.* On the Origin of Seebeck Coefficient Inversion in Highly Doped Conducting Polymers. *Adv. Funct. Mater.* **32**, 2112276 (2022).

17. Wakayama, Y. & Hayakawa, R. Antiambipolar Transistor: A Newcomer for Future Flexible Electronics. *Adv. Funct. Mater.* **30**, 1903724 (2020).
